# Outcomes for critical illness in children with cancer: Analysis of risk factors for adverse outcome and resource utilization from a specialized center in Mexico

**DOI:** 10.3389/fonc.2022.1038879

**Published:** 2022-11-30

**Authors:** Adolfo Cardenas-Aguirre, Montserrat Hernandez-Garcia, Berenice Lira-De-Leon, Yulissa L. Munoz-Brugal, Huiqi Wang, Ivonne Villanueva-Diaz, Eduardo Ruiz-Perez, Jose M. Mijares-Tobias, Alex O. Giles-Gonzalez, Jennifer McArthur, Gabriela Escamilla-Aisan, Anita Arias, Meenakshi Devidas, Asya Agulnik

**Affiliations:** ^1^ Department of Global Pediatric Medicine. St Jude Children’s Research Hospital, Memphis, TN, United States; ^2^ Department of Critical Care Medicine, Hospital Infantil Teleton de Oncologia, Queretaro, Mexico; ^3^ Pediatric Oncologist, MELOSA, Clinica Brugal, Santo Domingo, Dominican Republic; ^4^ Department of Epidemiology and Cancer Control, St Jude Children’s Research Hospital, Memphis, TN, United States; ^5^ Division of Critical Care Medicine, St Jude Children’s Research Hospital, Memphis, TN, United States; ^6^ Chief Medical Officer, Hospital Infantil Teleton de Oncologia, Queretaro, Mexico

**Keywords:** pediatric intensive care unit (PICU), onco-critical care, pediatric cancer, low- and middle-income countries (LMIC), outcomes, resource utilization

## Abstract

**Introduction:**

Children with cancer have a higher risk of adverse outcomes during critical illness than general pediatric populations. In Low- and middle-income countries, lack of resources can further negatively impact outcomes in critically ill children with cancer.

**Methods:**

In this study, we describe the outcomes of a large cohort of children with cancer including mortality and resource utilization. We performed a retrospective review of all patients admitted to our PICU between December 12th, 2013 and December 31st, 2019. Outcomes were defined as recovery or death and resource utilization was described via use of critical care interventions, Length of stay as well as PICU- and Mechanical Ventilation- free days.

**Results:**

Overall mortality was 6.9% while mortality in the unplanned admissions was 9.1%. This remained lower than expected mortality based on PIM2 scoring. Type of PICU admission, Neurological Deterioration as a cause of PICU admission, and PIM2 were significant as risk factors in univariate analysis, but only PIM2 remained significant in the multivariate analysis.

**Discussion:**

Our Study shows that high survival rates are achievable for children with cancer with critical illness in resource-limited settings with provision of high-quality critical care. Organizational and clinical practice facilitating quality improvement and early identification and management of critical illness may attenuate the impact of known risk factors for mortality in this population.

## Introduction

An estimated 400,000 children and adolescents are diagnosed with cancer every year worldwide (1). The burden of pediatric cancer is very high, with an estimated 11.1 million years-of-life-lost (YLL) in 2017, and this burden is disproportionately shifted towards low- and middle-income countries (LMICs) where unfortunately 90% of the cases occur (2). Up to 40% of these children experience critical illness and will require care in a pediatric intensive care unit (PICU) during the course of their cancer treatment (3, 4).

While the true burden of acute critical illness is unknown, previous point prevalence studies focusing on specific diseases suggest that at least 80% of the 64 million annual deaths in children take place in LMICs, where lack of resources can negatively impact the outcomes for acute critical illness (5, 6) and oncological disease (7). In High-income countries (HICs), children with cancer have a higher risk for adverse outcomes than general pediatric patients during critical illness, with mortality ranging from 6.8-27% (4), representing mortality almost three times higher than that of previously healthy children with critical illness (8). Furthermore, a recent meta-analysis found a 27.8% mortality rate for this population in HIC, with little change over the past 20 years (9).

Although there is limited data on outcomes of critical illness for children with cancer in LMICs, available studies report higher mortality (17-50%) for selected cohorts (10–12). For instance, a recent multi-site analysis describing characteristics of deterioration events in hospitalized children with cancer in Latin America found a mortality of 27% (13). However, more data is needed to better understand the outcomes of critical illness and prognostic factors for these patients in LMICs. Moreover, critical care resource utilization in this population has not been previously described in resource-limited settings, which is particularly relevant to adequately and effectively allocate available but limited resources in LMICs.

In this study, we describe the outcomes of a large cohort of children with cancer admitted to the PICU of a single specialized pediatric cancer center in Mexico and identified potential risk factors associated with adverse outcomes. We also aim to provide a description of resource utilization in this setting.

## Material and methods

### Setting

Hospital Infantil Teleton de Oncologia (HITO) is a dedicated pediatric cancer hospital located in central Mexico. It is the only dedicated pediatric cancer center in the country caring for children aged 0 to 18 years old (at the time of diagnosis) and is a national referral center. HITO is a comprehensive facility with a mixed private and public funding management scheme. It includes a 27-bed inpatient ward, a 4-bed dedicated PICU and a 4-bed Hematopoietic Stem Cell transplant (HSCT) unit, as well as a patient housing facility located at walking distance from the hospital.

### Data collection

We conducted a retrospective review of all patients admitted to our PICU between December 12^th^, 2013 and December 31^st^, 2019. Patients older than 18 years of age and those without a diagnosis of malignancy were excluded. In addition, patients transferred out of our PICU to another institution before resolution of their acute illness were excluded since critical illness outcomes could not be adequately followed. This study was approved by the Institutional Review Boards at HITO and St. Jude Children’s Research Hospital (SJCRH).

Patients were identified using the electronic PICU admissions and discharge log and clinical information was extracted from a retrospective review of electronic medical records using a case report form. Each patient was assigned a personal study ID number and likewise each admission event was assigned an admission ID number. The de-identified data was saved in MS Excel and used for data analysis.

### Definitions

Outcomes were defined as recovery or death (including death in the PICU or within 48 hrs. of PICU discharge). Patient characteristics included gender, age, type of malignancy, type of oncological treatment received prior to PICU admission, tumor activity (relapsed or refractory disease – defined as new or persistent tumoral activity after oncological treatment vs. all others), use of steroids prior to PICU admission (yes/no), mucosal barrier injury (defined by the Center of Diseases Control in the United States of America) (14), type of PICU admission (planned - defined as an elective admission that could potentially be delayed or cancelled without increasing the immediate risk of patient death or injury, e.g., scheduled surgical admissions, vs. unplanned – medical or other emergencies where the admission cannot be delayed), main cause for PICU admission, Pediatric index of mortality 2 (PIM2) score (15) on admission, use of mechanical ventilation (yes/no), use of renal replacement therapies (RRT), PICU length of stay, duration of mechanical ventilation, PICU free days within the first 30 days and mechanical ventilation free days within the first 30 days (defined as days where the patient was alive and free of the intervention during the first 30 days following the onset of their critical illness).

### Statistical analysis

Descriptive statistics were used to summarize characteristics, outcomes, and resource utilization for all PICU admissions identified. Chi-square test, Fisher Exact test, t-test or ANOVA were used to identify univariate risk factors for ICU mortality, as appropriate. Multivariable analysis of risk factors for mortality used a generalized estimating equation (GEE) model, controlling for multiple sampling (multiple ICU admissions for individual patients).

## Results

A total of 469 PICU admissions in 238 individual patients were identified during the 6 years of the study period. Of these, 1 was excluded because of age >18 years and an additional 8 were excluded because they did not have a cancer diagnosis. An additional patient was eliminated because he was transferred per guardians’ request to a different facility from our PICU before resolution of critical Illness, and no follow up data was available. This resulted in a final sample size of 459 admissions among 228 patients (mean 2.1 admissions/patient) used for analysis.

### Admission characteristics

Patient characteristics are summarized in **Table 1**. The most frequent causes of PICU admission were: Post-surgical admission (167, 36.4%), Sepsis (121, 26.4%), Respiratory distress (88, 19.2%), and Neurological deterioration (69, 15%). Other less common causes for PICU admission include Oncologic emergencies (24, 5.2%), Non-septic cardiovascular dysfunction (19, 4.1%), Coagulopathy, hemorrhage and/or anemia (14, 3.1%) and primary toxicity from drugs including chemotherapy (12, 2.6%). Some patients presented multiple causes for the same PICU Admission. Unplanned admissions represented 66.7% of all admissions (n=306).

**Table 1:** Summary of Patient Characteristics.

	Total
	(N=459)
**Age (years)**	
Mean (sd)	7.62 (5.24)
Median (Min, Q1, Q3, Max)	7 (0.04, 3.00, 12.00, 18.00)
**Gender, n(%)**	
Female	203 (44.2)
Male	256 (55.8)
**Type of PICU admission (Planned vs Unplanned, n(%)**	
Elective (planned)	153 (33.3)
Non-Elective (unplanned)	306 (66.7)
**PICU Diagnosis on admission, n=521(%)**	
Neurological Deterioration	69 (15.0)
Other	76 (16.6)
Respiratory distress	88 (19.2)
Sepsis	121 (26.4)
Major Surgery Post-operative admission	167 (36.4)
**Oncological disease group, n(%)**	
Central Nervous System Tumor	112 (24.4)
Hematological Malignancy	204 (44.4)
Solid tumor (outside CNS)	143 (31.2)
**Outcome, n(%)**	
Death	32 (7.0)
Survival	427 (93.0)
**PIM2 (%)**	
Mean (SD)	7.24 (13.15)
Median (Min, Q1, Q3, Max)	2.6 (0.05, 1.10, 7.90, 94.10)
**Total ICU stay (days)**	
Mean (SD)	9.04 (11.35)
Median	5
Min, Q1, Q3, Max	1.00, 3.00, 9.00, 89.00
**Mechanical Ventilation (Yes or No), n(%)**	
No	257 (56.0)
Yes	202 (44.0)
**Total days with mechanical ventilation (Among Yes)**	
Mean (SD)	9.35 (11.12)
Median (Min, Q1, Q3, Max)	5 (1.00, 2.00, 12.00, 79.00)

### Outcomes and risk factors for mortality

Thirty-two patients died during their PICU stay or within the first 48 hours after discharge, for a mortality of 6.9%. This was higher for unplanned admissions (28/306, 9.1%.) than for planned admissions (4/153, 2.3%, p=0.0104). Of the 32 deaths, 1 (3%) patient death occurred within 24 hours, 6 (18.7%) within the first 48 hours and 9 (29.1%) within the first 72 hours of PICU admission. Most PICU deaths occurred before 21 days of admission, accounting for 28 of the 32 deaths (87.5%).

The observed mortality was similar to the expected mean mortality for all admissions as predicted by the PIM2 (6.9% vs 7.2% respectively) and for the unplanned admissions group (9.1% vs 9.7%). When analyzing mortality by quartiles for ‘all admissions’ and ‘unplanned admissions only’, the observed mortality was higher in the lower risk groups (Q1 and Q2), similar in the Q3, and lower in the sickest patients (Q4) (**Supplementary Table 1**).

In the univariate analysis, type of PICU admission, Neurological Deterioration as a cause of PICU admission, and PIM2 were the only risk factors at admission associated to mortality (**Table 2**). Notably, of the 202 PICU admissions requiring mechanical ventilation, 14.9% (30/202) resulted in mortality, and for the 20 admissions requiring RRT, 55% (11/20) resulted mortality. In our multivariate analysis, only PIM2 was an independent risk factor for mortality (See **Table 3**); when this was removed from the model, no other factors reached significance, though Neurological deterioration as an admission diagnosis had a trend towards higher mortality (See **Table 4**). This was similar in our analysis focused only on unplanned admissions (**Supplementary Table 2**).

**Table 2:** Univariate analysis of risk factors for mortality (GEE MODEL) among all admissions.

Factor	Category	All Admissions (n=459)		Univariate Analysis	
		Survivors N (%)	Non-Survivors N(%)	P-value	Odds Ratio
Type of PICU admit	Non-Elective (Unplanned)	278 (90.8)	28 (9.2)	0.0022	3.75 (1.29 - 10.88)
	Elective (Planned)	149 (97.4)	4 (2.6)		1.00 (ref)
Neurological Deterioration as cause of PICU admission	Yes	59 (85.5)	10 (14.5)	0.0446	2.84 (1.32 - 6.12)
	No	368 (94.4)	22 (5.6)		1.00 (ref)
Respiratory distress as cause of PICU admission	Yes	79 (89.8)	9 (10.2)	0.2392	1.74 (0.78 - 3.88)
	No	348 (93.8)	23 (6.2)		1.00 (ref)
Sepsis as cause of PICU Admission	Yes	108 (89.3)	13 (10.7)	0.0933	2.02 (0.97 - 4.19)
	No	319 (94.4)	19 (5.6)		1.00 (ref)
Type of Malignancy	CNS tumor	103 (92.0)	9 (8.0)	0.0743	2.43 (0.82 - 7.19)
	Hematological Malignancy	186 (91.2)	18 (8.8)		2.69 (1.00 - 7.29)
	Solid tumor (outside CNS)	138 (96.5)	5 (3.5)		1.00 (ref)
Oncologic treatment prior to PICU admission	HSCT	11 (100.0)	0		
	Low toxicity treatment	54 (93)	4 (7)	0.3696	1.48 (0.37 - 5.98)
	Myelotoxic chemotherapy	181 (93.3)	13 (6.7)		1.47 (0.51 - 4.24)
	None (New Diagnosis)	77 (88.5)	10 (11.5)		2.73 (0.91 - 8.21)
	Surgery	104 (95.4)	5 (4.6)		1.00 (ref)
Tumor activity	Relapsed or refractory disease	61 (91.0)	6 (9.0)	0.5277	1.39 (0.55 - 3.52)
	All others	366 (93.3%)	26 (6.7%)		1.00 (ref)
Steroids prior to PICU admission	No	252 (92.3)	21 (7.7)	0.4340	1.34 (0.63 - 2.84)
	Yes	175 (94.1)	11 (5.9)		1.00 (ref)
PIM2	Mean (median)	6% (2.4%)	23.9% (8.55%)	0.0040*	1.05 (1.03 - 1.07)
Mucosal barrier injury	No	318 (93.8)	21 (6.2)	0.3148	0.65 (0.30 - 1.42)
	Yes	109 (90.8)	11 (9.2)		1.00 (ref)

**Table 3:** Multivariate analysis of Risk Factors for Mortality (GEE Model) among all admissions (including PIM2).

Factor	Category	Multivariable Analysis	
		P-value	Odds Ratio
Type of PICU admit	Non-Elective (Unplanned)	0.0433	2.75 (0.91 - 8.30)
	Elective (Planned)		1.00 (ref)
Neurological Deterioration as cause of PICU admission	Yes	0.1914	2.21 (0.77 - 6.35)
	No		1.00 (ref)
PIM2		0.0068	1.05 (1.03 - 1.07)

**Table 4:** Multivariate analysis of Risk Factors for Mortality (GEE Model) among all admissions (NOT including PIM2).

Factor	Category	Multivariable Analysis	
		P-value	Odds Ratio
Type of PICU admit	Non-Elective (Unplanned)	0.4235	1.82 (0.42 - 7.93)
	Elective (Planned)		1.00 (ref)
Neurological Deterioration as cause of PICU admission	Yes	0.0966	2.94 (0.94 - 9.21)
	No		1.00 (ref)
Sepsis	Yes	0.1207	2.05 (0.83 - 5.07)
	No		1.00 (ref)
Type of Malignancy	CNS tumor	0.4512	1.74 (0.54 - 5.60)
	Hematological		1.84 (0.62 - 5.48)
	Solid tumor (outside CNS)		1.00 (ref)

### Resource utilization

Overall, 202 admissions (44%) required mechanical ventilation and of these 132 were unplanned admissions (n= 306, 43%). Mean and median duration of mechanical ventilation was 9.35 and 5 days respectively (range of 1-79 days). Twenty patients (4.4%) required renal replacement therapy alone or in combination with other extracorporeal depuration techniques (3 of these patients received plasmapheresis and RRT simultaneously), and 5 patients (1.1%) received other extracorporeal depuration therapies without RRT (4 patients received plasmapheresis and 1 leukapheresis). The mean and median PICU Length of stay were 9.04 and 5 days respectively (range 1-89 days).

Neither duration of mechanical ventilation (among those who received it) or length of stay (among all patients) were significantly different among survivors and non-survivors (p=0.96 and 0.60, respectively; See **Supplementary Table 3**). When analyzing all admissions, hematological malignancies were associated with both an increased PIM2 score and less mechanical ventilation-free and PICU-free days (**Supplementary Table 4**). However, when focusing only on unplanned admissions, hematological malignancies were only associated with higher disease severity (PIM2 score) and not with increased resource utilization (mechanical ventilation-free or PICU-free days.) (**Supplementary Table 5**).

**Figure 1:**
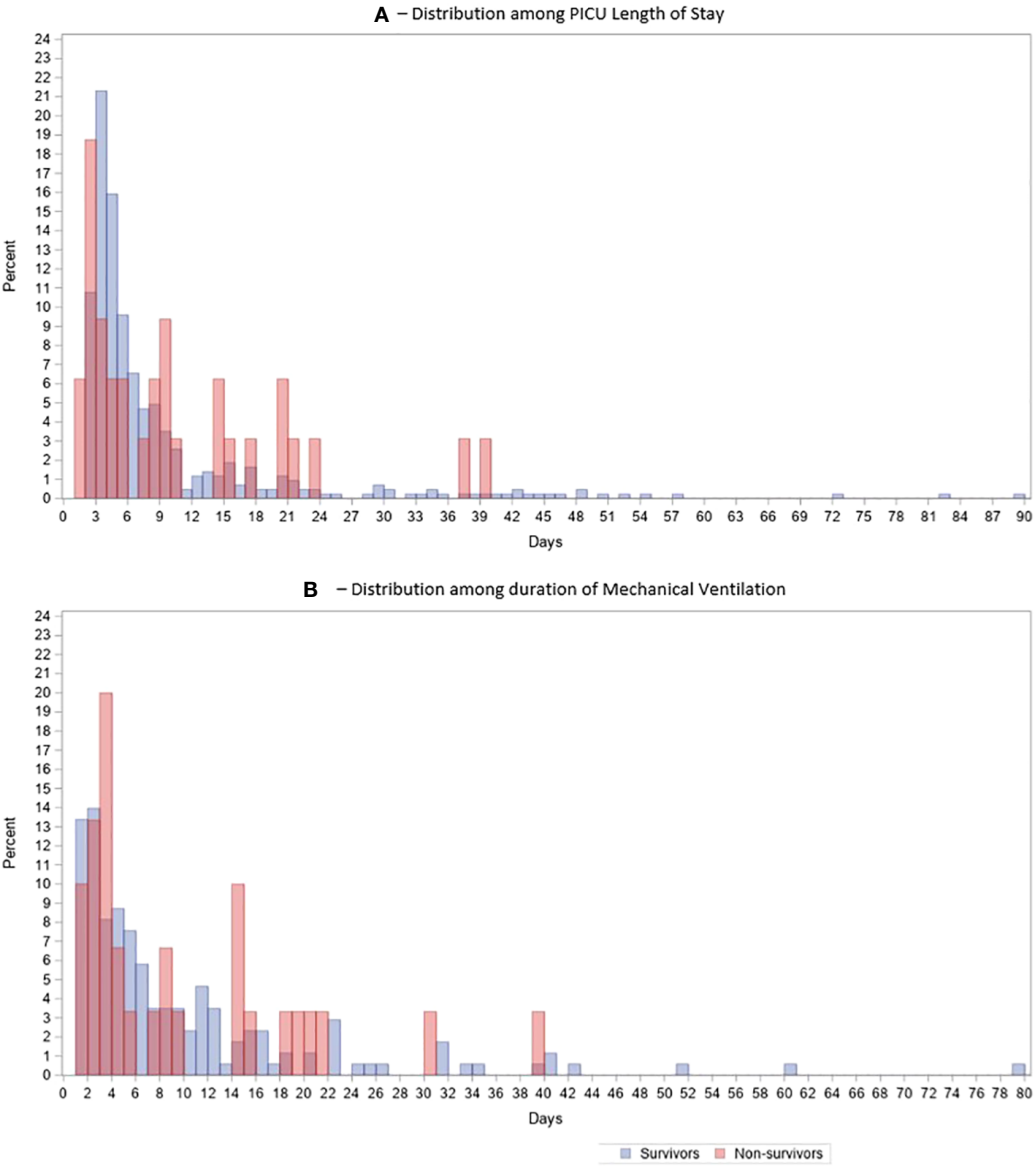
Histogram Distribution of Survivors vs Non-survivors over time, **(A)** Distribution over days of PICU stay, **(B)** Same distribution over duration of Mechanical Ventilation.

Seventy-six (16.5%) patients had a prolonged PICU length of stay (defined as LOS > 14 days) with 27 patients having a PICU LOS greater than 30 days (5.8%). Of note, survival for these admissions was 89.5% (68/76 patients) for the group with LOS > 14 days and 92.6% (25/27 patients) for the group with LOS > 30 days (**Figure 1**).

Out of 202 admissions, 40 patients (19.8%) who required mechanical ventilation (MV) needed prolonged mechanical ventilatory support (longer than 14 days), and 13 (6.4%) required mechanical ventilation for more than 30 days. Survival for the > 14 days MV group was 82% (33/40) and 92% (12/13) for the more than 30 days MV group.

## Discussion

Our study of PICU admissions at a dedicated pediatric oncology hospital in Mexico over a period of 6 years found a lower mortality rate (9.1%) for unplanned admissions than previously described in LMIC (between 27% and 77% (13, 16, 17). Despite being a resource-limited hospital in an upper-middle income country, this is comparable to reported mortality rates in HIC between 6.8% to 17.5% (4, 18). These findings highlight the fact that it is possible to attain high survival rates for critically ill children with cancer in resource-limited settings.

The main causes for PICU admission in this cohort were consistent with published literature, including planned post-surgical admissions and unplanned admissions for neurological deterioration, respiratory distress, and sepsis (3, 19). In our study, the only characteristics at admission identified as significant risk factors were severity of illness (PIM2) score and unplanned admission, similar to prior studies (9, 13). The distinction between planned and unplanned PICU admissions is important, since planned/post-surgical admissions make up the majority of oncology PICU admissions and have a significantly lower risk of mortality. Thus, further studies seeking to improve outcomes for critically ill children with cancer should focus on unplanned or emergency admissions and hospitalized patients with deterioration events, which represent the majority of adverse outcomes and mortality.

In previous studies, the need for mechanical ventilation (20) and renal replacement therapy (4, 9, 21) during the PICU stay have been associated with poor survival. This finding was confirmed in our study population, where outcomes for children requiring mechanical ventilation and RRT were similar to those reported on HIC, (MV mortality rate of 14.8% in our population vs 15-40% in reported literature (4, 9) and a mortality of 55% for those requiring RRT vs 54.5% in published literature (22).

Most deaths in this cohort occurred before 21 days of PICU stay, with longer PICU admissions having relatively high survival rates. These prolonged-stay admissions included patients with central nervous tumors or hematological malignancies and multiple PICU reasons for PICU admission including a combination of sepsis, respiratory distress, coagulopathy and/or neurological deterioration. Patients in this subgroup required prolonged stays for rehabilitation and weaning or subsequent myelotoxic chemotherapy after resolution of the primary event with potential for additional toxicity-related complications. Encouragingly, the survival rates for these long-stay patients are similar in our study to those described in the literature for all unplanned PICU admissions (4, 9) and higher than that described for prolonged-stay in general PICU admissions(95.2% vs 80%) (23). This exemplifies the fact that despite risk factors, many children with cancer who experience critical illness can recover with adequate supportive care. This finding is an important divergence from the common belief that many of these patients will not survive critical illness in resource-limited settings, leading to inadequate resource and ultimately poor outcomes (8).

Our center’s relatively low mortality in critically ill children with cancer compared to other resource-limited settings is likely due to a combination of factors and practices that may improve outcomes in these patients. As a dedicated pediatric cancer center, we have systematically implemented a number of quality practices intended to improve care for this patient population, including: a) training and education of the clinical staff managing critical illness in the child with cancer b) early identification of deterioration events facilitated by a Pediatric Early Warning System (PEWS) (12) validated in oncology patients (12, 24), c) timely PICU transfer of deteriorating patients due to a proactive critical care outreach team and our favorable ratio of critical care beds to regular floor beds leading to few PICU-level interventions performed on the ward; d) Rapid access to clinical care for outpatients in the nearby housing facility, e) Implementation of a Golden Hour initiative for antibiotic administration in febrile neutropenia, among others. Consequently, our center’s lower mortality rates support prior work demonstrating that simple organizational and clinical interventions can lead to significant improvement in outcomes for these patients in centers of all resource-levels (13)

Notably, more than half our PICU patients did not require mechanical ventilation, even in the unplanned admission group (43%), which is similar to that reported in previous studies, including a large multicenter cohort in Latin America (48-53%%) (13, 20) We interpret this as a marker of proactive identification of deterioration events and timely PICU transfer of patients with critical illness. While it may be argued that some of these admissions do not actually experience critical illness, our expected mortality is similar to that of a large Argentinian cohort (25). Similarly, our observed mortality and performance (observed/expected mortality) in the unplanned admissions, mechanical ventilation and RRT subgroups is comparable to that of high-resource settings. Early intervention before the need for invasive mechanical ventilation may lead to resolution of critical illness through early institution of non-invasive respiratory support, vasoactive infusions, or extracorporeal purifying therapies such as plasmapheresis, leukapheresis or conventional renal replacement therapies. Early institution of continuous multisystem monitoring only available in an PICU setting may also improve our ability to detect deterioration, allowing for earlier intervention and resolution of critical illness in these high-risk patients.

There are some limitations to our study. First, this is a single center cohort from a hospital specializing in the care of children with cancer and our results may not be generalizable to all resource-limited hospitals. Also, the retrospective nature of our study limited our data analysis to that available in the patients’ charts; data on the use of vasoactive infusions and organ dysfunction scores were unavailable. The relatively low mortality in our study may also have prevented identification of significant risk factors for mortality due to power limitations. Despite these limitations, we included all eligible admissions and had no exclusions due to incomplete data, and this study represents one of the largest cohorts of pediatric oncology patients with critical illness in a hospital in Latin America. Our study’s demonstrated low mortality represents an important addition to the literature and highlights the impact of dedicated expertise and prioritization of this high-risk patient population despite resource limitations.

## Conclusion

High survival rates for children with cancer with critical illness are achievable in resource-limited settings with provision of high-quality critical care. As exemplified in our study, organizational and clinical practice facilitating quality improvement and early identification and management of critical illness may attenuate the impact of known risk factors for mortality in this population. Future collaborative studies in different regions and hospital resource levels should be aimed at evaluating the impact of these interventions to improve outcomes for children with cancer globally.

## Data availability statement

The data analyzed in this study is subject to the following licenses/restrictions: De-identified datasets are available upon requests to the authors. Original data (codebreaker) is derived from patient charts and contains patient information (PHI), thus it is not available for sharing, only available to collaborators at the study site. Requests to access these datasets should be directed to AC-A - adolfo.cardenas-aguirre@stjude.org.

## Author contributions

AC-A, conceptualization, methodology, data entry and cleaning, and writing original draft. MH-G, conceptualization, methodology – variable design and definitions, writing review, and editing. BL-D-L, data entry, data cleaning, writing review, and editing. YM-B, methodology, data entry, writing review, and editing. HW, methodology – statistical analysis, and data cleaning. IV-D, ER-P, JM-T, and AG-G, data entry, writing review, and editing. JM, conceptualization, writing review, and editing. GE-A, supervision, writing review, and editing. AAr, writing review, and editing. MD, conceptualization, methodology – statistical analysis, writing review, and editing. AAg, conceptualization, methodology, supervision, writing review, and editing. All authors contributed to the article and approved the submitted version.

## Acknowledgments

The authors would like to acknowledge the support provided by authorities and staff at Hospital Infantil Teleton de Oncologia for the support in conducting this research, as well as the support provided by the Department of Global Pediatric Medicine at St Jude Children’s Research Hospital.

## Conflict of interest

The authors declare that the research was conducted in the absence of any commercial or financial relationships that could be construed as a potential conflict of interest.

## Publisher’s note

All claims expressed in this article are solely those of the authors and do not necessarily represent those of their affiliated organizations, or those of the publisher, the editors and the reviewers. Any product that may be evaluated in this article, or claim that may be made by its manufacturer, is not guaranteed or endorsed by the publisher.
