# Supplementary material for: Outcomes for critical illness in children with cancer: Analysis of risk factors for adverse outcome and resource utilization from a specialized center in Mexico
Source: Front Oncol. 2022 Nov 30;12:1038879. doi: 10.3389/fonc.2022.1038879 (PMC9748566; doi:10.3389/fonc.2022.1038879)
Supplement: Supplementary file 1 [file DataSheet_1.docx]

Supplementary Tables

# Supplementary Tables

SUPPLEMENTAL TABLE 1 – Predicted vs Observed Mortality Across Quartiles.

| All ADMISSIONS (N=459) | | | | | | |
| --- | --- | --- | --- | --- | --- | --- |
| Risk Group (Q) | Max. Probability Of Death | N | Predicted Mortality | Observed Mortality | Predicted Deaths | Observed Deaths |
| 1 | 1.1 | 120 | 0.51% | 1.67% | 0.612 | 2 |
| 2 | 2.7 | 118 | 1.86% | 5.08% | 2.1948 | 6 |
| 3 | 8 | 112 | 5.12% | 6.25% | 5.7344 | 7 |
| 4 | 94.1 | 109 | 22.60% | 15.60% | 24.634 | 17 |
|  |  |  |  |  |  |  |
| UNPLANNED ADMISSIONS (N=306) | | | | | | |
| Risk Group (Q) | Max. Probability Of Death | N | Predicted Mortality | Observed Mortality | Predicted Deaths | Observed Deaths |
| 1 | 1.9 | 77 | 1.03% | 3.90% | 0.7931 | 3 |
| 2 | 5.3 | 77 | 3.28% | 9.09% | 2.5256 | 7 |
| 3 | 10.35 | 76 | 7.84% | 7.89% | 5.9584 | 6 |
| 4 | 94.1 | 76 | 27.27% | 15.79% | 20.7252 | 12 |

Max- Maximum.

SUPPLEMENTAL TABLE 2 Univariate analysis of risk factors for mortality (GEE MODEL) Unplanned admissions only.

| Factor | Category | Unplanned Admissions N=306  Univariate Analysis | | | |
| --- | --- | --- | --- | --- | --- |
|  |  | Survivors N (%) | Non-Survivors (N, %) | P-value | Odds Ratio |
| Age |  | 8.0 (7.5) | 6.8 (5.0) | 0.2618 | 0.96 (0.88 - 1.04) |
| Gender | F | 116 (89.2) | 14 (10.8) | 0.3980 | 1.41 (0.65 - 3.04) |
|  | M | 162 (92.0) | 14 (8.0) |  | 1.00 (ref) |
| Neurological Deterioration as PICU admission cause | Yes | 57 (86.4) | 9 (13.6) | 0.2018 | 1.84 (0.81 - 4.19) |
|  | No | 221 (92.1) | 19 (7,9) |  | 1.00 (ref) |
| Respiratory distress as PICU admission cause | Yes | 78 (89.7) | 9 (10.3) | 0.6488 | 1.22 (0.53 - 2.80) |
|  | No | 200 (91.3) | 19 (8.7) |  | 1.00 (ref) |
| Sepsis as PICU admission cause | Yes | 106 (89.1) | 13 (10.9) | 0.4022 | 1.40 (0.65 - 3.04) |
|  | No | 172 (92.0) | 15 (8.0) |  | 1.00 (ref) |
| Type of Malignancy | CNS tumor | 44 (86.3) | 7 (13.7) | 0.3335 | 2.50 (0.74 - 8.44) |
|  | Hematological Malignancy | 171 (91.0) | 17 (9.0) |  | 1.55 (0.52 - 4.63) |
|  | Solid tumor (outside CNS) | 63 (94.0) | 4 (6.0) |  | 1.00 (ref) |
| Oncologic treatment prior to PICU admission | HSCT | 11 (100.0) | 0 (0.0) |  |  |
|  | Low toxicity treatment | 36 (90.0) | 4 (10.0) | 0.3890 | 0.78 (0.16 - 3.87) |
|  | Myelotoxic chemo | 156 (92.9) | 12 (7.1) |  | 0.54 (0.14 - 2.02) |
|  | None | 53 (85.5) | 9 (14.5) |  | 1.27 (0.33 - 4.94) |
|  | Surgery | 22 (88.0) | 3 (12.0) |  | 1.00 (ref) |
| Tumor activity | Relapsed or Refratory disease | 30 (83.3) | 6 (16.7) | 0.1914 | 2.25 (0.83 - 6.10) |
|  | All others | 248 (91.9) | 22 (8.1) |  | 1.00 (ref) |
| Steroids prior to PICU admission | No | 148 (89.7) | 17 (10.3) | 0.4120 | 1.00 (ref) |
|  | Yes | 130 (92.2) | 11 (7.8) |  | 0.72 (0.33 - 1.57) |
| PIM2 |  | 8.3% (4.5%) | 23.8% (9.1%) | ***0.0141**** | 1.04 (1.02 - 1.06) |
| Mucosal barrier injury | No | 170 (90.4) | 18 (9.6) | 0.7437 | 1.00 (ref) |
|  | Yes | 108 (91.5) | 10 (8.5) |  | 0.87 (0.38 - 1.99) |
| Order of admission | 1st | 75 (82.4) | 16 (17.6) | 0.2094 | 1.00 (ref) |
|  | 2nd | 81 (96.4) | 3 (3.6) |  | 0.42 (0.12 - 1.41) |
|  | 3rd | 33 (91.7) | 3 (8.3) |  | 1.07 (0.30 - 3.75) |
|  | 4+ | 89 (93.7) | 6 (6.3) |  | 1.76 (0.62 - 5.00) |

PICU = Pediatric Intensive Care Unit. PIM2 – Pediatric Index of Mortality. HSCT Hematopoietic stem cell transplant. Ref= Reference.

SUPLEMENTAL TABLE 3 Resource utilization in survivors vs non survivors.

|  | *Total* | *Survivors* | *Non Survivors* | *P-value* |
| --- | --- | --- | --- | --- |
|  | *(N=459)* | *(N=427)* | *(N=32)* |  |
| **Total PICU stay (days)** | | | |  |
| Mean (SD) | 9.04 (11.35) | 8.97 (11.46) | 10.03 (9.84) | 0.6095^a^ |
| Median | 5 | 5 | 7.5 |  |
| Min, Q1, Q3, Max | 1.00, 3.00, 9.00, 89.00 | 2.0, 3.0, 9.0, 89.0 | 1.0, 2.5, 14.5, 39.0 |  |
| **Total days with mechanical ventilation (Among Yes)** | | | |  |
| Mean (SD) | 9.35 (11.12) | 9.36 (11.41) | 9.27 (9.42) | 0.9661^a^ |
| Median | 5 | 5 | 4.5 |  |
| Min, Q1, Q3, Max | 1.00, 2.00, 12.00, 79.00 | 1.0, 2.0, 11.5, 79.0 | 1.0, 3.0, 14.0, 39.0 |  |
| **Mechanical ventilation free days (first 30 days)** | | | |  |
| Mean (SD) | 24.79 (9.12) | 26.54 (6.72) | 1.47 (2.06) | <.0001^a^ |
| Median | 30 | 30 | 1 |  |
| Min, Q1, Q3, Max | 0.00, 24.00, 30.00, 30.00 | 0.0, 26.0, 30.0, 30.0 | 0.0, 0.0, 2.0, 8.0 |  |
| **PICU free days (first 30 days)** | | | |  |
| Mean (SD) | 20.29 (9.40) | 21.81 (7.88) | 0.09 (0.39) | <.0001^a^ |
| Median | 25 | 25 | 0 |  |
| Min, Q1, Q3, Max | 0.00, 17.00, 27.00, 28.00 | 0.0, 21.0, 27.0, 28.0 | 0.0, 0.0, 0.0, 2.0 |  |
| ^a^ T-test; | | | |  |

PICU Pediatric Intensive Care Unit. Min Minimum. Max, Maximum. SD standard Deviation. Q1,

SUPLEMENTAL TABLE 4 Association of type of malignancy with severity of critical illness and resource utilization among all PICU admissions

| Variable | Oncological disease group | N (%)  (N=459) | Mean (std) | P-value^1^ |
| --- | --- | --- | --- | --- |
| PIM2 (%) | CNS tumor | 112 (24.4) | 3.77 (5.56) | <.0001 |
|  | Hematological Malignancy | 204 (44.4) | 10.87 (15.29) |  |
|  | Solid tumor (outside CNS) | 143 (31.2) | 4.78 (12.85) |  |
|  |  |  |  |  |
| Mechanical ventilation free days (first 30 days) | CNS tumor | 112 (24.4) | 23.51 (9.35) | 0.006 |
|  | Hematological Malignancy | 204 (44.4) | 24.10 (9.81) |  |
|  | Solid tumor (outside CNS) | 143 (31.2) | 26.79 (7.48) |  |
|  |  |  |  |  |
| PICU free days (first 30 days) | CNS tumor | 112 (24.4) | 19.61 (9.84) | 0.009 |
|  | Hematological Malignancy | 204 (44.4) | 19.27 (9.76) |  |
|  | Solid tumor (outside CNS) | 143 (31.2) | 22.29 (8.20) |  |
| ^1^ ANOVA | | | | |

PICU Pediatric intensive Care Unit. PIM2 Paediatric index of mortality 2. CNS Central nervous system.

SUPLEMENTAL TABLE 5. Association of type of malignancy with disease severity and resource utilization (Unplanned admissions only)

| Variable | Oncological disease group | N (%)  (N=306) | Mean (std) | P-value^1^ |
| --- | --- | --- | --- | --- |
| PIM2 (%) | CNS tumor | 51 (16.7) | 6.29 (7.14) | 0.0163 |
|  | Hematological Malignancy | 188 (61.4) | 11.62 (15.67) |  |
|  | Solid tumor (outside CNS) | 67 (21.9) | 7.04 (15.34) |  |
|  |  |  |  |  |
| Mechanical ventilation free days (first 30 days) | CNS tumor | 51 (16.7) | 21.51 (10.08) | 0.1263 |
|  | Hematological Malignancy | 188 (61.4) | 24.10 (9.93) |  |
|  | Solid tumor (outside CNS) | 67 (21.9) | 25.16 (9.53) |  |
|  |  |  |  |  |
| PICU free days (first 30 days) | CNS tumor | 51 (16.7) | 16.86 (10.55) | 0.0899 |
|  | Hematological Malignancy | 188 (61.4) | 19.30 (9.82) |  |
|  | Solid tumor (outside CNS) | 67 (21.9) | 20.88 (9.29) |  |
| ^1^ ANOVA | | | | |

PICU Pediatric intensive Care Unit. PIM2 Paediatric index of mortality 2. CNS Central nervous system.
